# Determinants of Residential Satisfaction During the Initial Stage of the COVID-19 Pandemic: The Case of Xiangyang City, China

**DOI:** 10.3389/ijph.2023.1606016

**Published:** 2023-11-28

**Authors:** Dinghuan Yuan, Haoyuan Yu, Wenyi Lin, Lisi Zha

**Affiliations:** ^1^ School of Public Administration and Emergency Management, Jinan University, Guangzhou, China; ^2^ Public Policy Institute, Jinan University, Guangzhou, China; ^3^ Common Prosperity and National Governance Institute, Jinan University, Guangzhou, China; ^4^ School of Public Administration, Guangdong University of Finance and Economics, Guangzhou, China

**Keywords:** character strengths, self-efficacy, social capital, residential satisfaction, COVID-19 pandemic

## Abstract

**Objectives:** To explore the impacts of psychological character strengths, self-efficacy, and the number of confirmed COVID-19 cases on residential satisfaction at the initial stage of the COVID-19 pandemic in China.

**Methods:** To achieve the study aim, we collected data from 281 observations from Xiangyang City, China, via an online survey. Data were analyzed using linear regression.

**Results:** Character strengths and the number of confirmed COVID-19 cases significantly impacted residential satisfaction. While self-efficacy did not directly impact residential satisfaction, it moderated the relationship between the number of confirmed COVID-19 cases and residential satisfaction. The control variables of social trust and shared value positively impacted residential satisfaction, and their influence on residential satisfaction was higher than that of character strengths. The sociodemographic variables of marriage, age, educational attainment, and housing area *per capita* also impacted residential satisfaction significantly. However, strong ties and weak ties became insignificant variables due to social distancing strategies.

**Conclusion:** The study findings offer insights for local governments to enhance residential satisfaction in the community to avoid social panic during unpredictable threats or future pandemics.

## Introduction

Although the pandemic occurred 3 years ago, it is still valuable to revisit this topic as we are in a high-risk society. Similar health crises can happen again. We still remember that several confirmed COVID-19 cases were reported in Wuhan, Hubei Province, China from mid-December 2019. When General Secretary Xi Jinping investigated and guided preventive measures to overcome COVID-19 in Beijing, he emphasized that community management is the first and most effective line of defense to prevent the community and international spread of the virus. On 27 January 2020, the Xiangyang City government implemented an unprecedented “closed city” policy to curb the spread of COVID-19. Xiangyang City, with its history of more than 2,800 years as the transportation hub of Hubei Province and a population of 6.05 million, was shut down. Local citizens could not leave the city; all airports, railway stations, bus stations, and highways were closed; all restaurants and public entertainment venues were shut down; all public activities were canceled; urban traffic was controlled; and many communities were isolated.

Quarantine methods such as self-isolation at home and social distancing were considered the most efficient ways to prevent the spread of COVID-19 infection during this period [1]. However, the lockdown strategy changed the residents’ daily lives unprecedentedly. The outbreak itself and the subsequent control measures led to widespread fear and panic, especially in communities with confirmed COVID-19 cases. The increasing number of reports of confirmed cases and deaths led to residents avoiding contact with their neighbors as they feared that they would get infected and subsequently die or lose loved ones. Fear and stress were also higher during the initial COVID-19 outbreak period because effective medicines and vaccines were not invented during this period [2]. Communities with confirmed COVID-19 cases usually adopt stricter restrictions on social contact and activities. These isolation strategies led to the residents and students adapting to remote work and learning via new technologies [3]. Psychological problems arose when people dealt with family issues while working from home. Social isolation and limited physical activity generate interpersonal alienation [4]. Some studies have revealed that a lack of social support and a change in lifestyle may be more threatening to an individual’s wellbeing than the risk of COVID-19 infection [5]. Moreover, the spread of COVID-19 and its control measures are associated with significant mental health problems, such as anxiety, fear, depressive symptoms, sense of loneliness, sleep disturbances [6, 7], and fatigue [8]. COVID-19 also caused psychological stress in infected patients and their families. Fear and panic about COVID-19 led to the infected patients and their families experiencing stigmatization and social exclusion [9, 10].

Therefore, the COVID-19 pandemic threatens individuals’ physical health and causes psychological distress [11], affecting their quality of life. Some studies have revealed that the pandemic and its control measures cause people to experience more negative emotions [12], consequently impacting their wellbeing and life satisfaction [13, 14]. Residential satisfaction serves as a criterion for judging the quality of life and psychological wellbeing [15, 16]. Residential satisfaction contributes to “social cohesion,” which plays an essential role in maintaining societal stability [17]. Understanding residential satisfaction with respect to urban livability in China and its determinants is beneficial for urban planning and policymaking [18]. Under the lockdown policy, residents’ daily activities changed, and their living spaces became restricted to the housing area during quarantine. The COVID-19 pandemic induced feelings of fear and panic among individuals toward their neighbors, especially in communities with confirmed cases. The previously constructed shared values and trust in the communities might have changed. When faced with an unpredictable threat, different individuals often suffer different extents of mental distress. Some studies revealed that individuals with high levels of character strengths would be able to select, alter, and implement their resources to decrease mental distress [19, 20]. Individuals with high self-efficacy are at a low risk of experiencing depression [21]. Thus, the effects of character strengths and psychological self-efficacy on residential satisfaction may have been strengthened to decrease mental distress during the COVID-19 pandemic. However, most studies ignored the impact of psychological factors on residential satisfaction under the particular conditions of health crisis. The abovementioned relationship remains unknown during the initial stage of the COVID-19 pandemic, but they are essential for policymakers to design policies to enhance residential satisfaction when facing the future pandemic. Against this background, this study analyzed this topic by using 281 observations collected from Xiangyang City, Hubei Province, China. General sociodemographic factors should not be ignored when considering the impacts of psychological factors on residential satisfaction during self-isolation at home. Therefore, this study also considered the social capital and sociodemographic predictors, such as, housing area, age, gender and educational attainment when focuses on the factors of character strengths and self-efficacy. Mental health problems caused by the overwhelming number of confirmed COVID-19 patients in the community are also considered in the context of COVID-19 in this study. We assume that residential satisfaction is positively related to character strengths and psychological self-efficacy and negatively correlated with the number of confirmed cases of COVID-19 in a given community.

## Methods

### Study Area and Population

Most residents live in urban communities due to the rapid urbanization process in China. This study focused on communities in an urban area of Xiangyang City, Hubei Province, which experienced the rapid spread of the COVID-19 virus. Given the lockdown policy implemented in Xiangyang City, we could not randomly distribute paper questionnaires to urban communities. Therefore, we disseminated an online-based questionnaire through the community WeChat group from February 2020 to March 2020 (Supplementary Material S1). The surveyed communities were mainly located in five communities: TXH, ZGC, SJQ, WF, and TFS. These five communities have been constructed for at least 5 years, indicating that they are mature. Members of these communities have been in contact for a long period. This connection can accurately test whether social networks influence residential satisfaction. The surveyed communities not only included residents in commercial residential buildings but also included family members living in a building allocated by the work unit, such as Yucai and Yi’an in the ZGC Community, with a history of more than 20 years. The mixed communities indicated that respondents from the five surveyed communities were from all walks of life.

### Data Collection

A total of 327 questionnaires were collected. After excluding the missing values, 281 valid questionnaires were selected. Overall, the sample can represent the population in the urban area of Xiangyang City during the initial stage of the COVID-19 pandemic to a large extent. However, considering that the survey was conducted online, we could not distribute the questionnaire evenly in each community. Most respondents lived in SJQ and TXH communities, accounting for 77% of the 281 questionnaires (Supplementary Material S2).

Residential satisfaction was used as the dependent variable in this study. The survey was conducted online during the initial outbreak period of COVID-19. Two questions were asked to the respondents to score their residential satisfaction regarding the hedonistic and eudaimonic components of wellbeing. Hedonism refers to a life of pleasure [22], while eudemonia focuses on living life in a full and deeply satisfying way [23]. These questions were as follows: “In general, how often do you feel joyful when you live in your community?” (S1), and “In general, to what extent do you feel contented when you live in your community?” (S2). The answers to the two questions were rated on a 5-point Likert scale ranging from 1 to 5, with a higher score indicating higher residential satisfaction.

This study added the number of confirmed cases of COVID-19, psychological self-efficacy, and character strengths as explanatory variables. The outbreak of the COVID-19 pandemic is known to disturb societal psychology [2]. COVID-19 also caused residents’ psychological states to change, further impacting their residential satisfaction. The respondents were asked to report the number of confirmed COVID-19 cases in their communities. Self-efficacy is a type of psychological self-suggestion that refers to the degree and intensity of one’s inner belief in completing a task or achieving a goal in a specific situation that may contain novel, unpredictable, and possibly stressful features [24, 25]. The measurement of psychological self-efficacy mainly referred to Wang and others’ study to evaluate residents’ self-efficacy in the particular condition of the COVID-19 pandemic [21]; the items included: “I believe I can avoid the infection” (SE1), “I know how to avoid the infection” (SE2), and “I believe that even if I were infected, I could be cured” (SE3).

Character strengths are defined as positive psychological traits that are reflected in thoughts, feelings, and behaviors. Character strengths are psychological processes that define six virtues [26]. However, most previous studies could not demonstrate the six-factor virtue structure theoretically postulated by Peterson and Seligman [26]. For instance, five-factor [27, 28], four-factor [29], and three-factor virtue structures [30] were established based on samples from different cultures. The consistent finding is that the strengths of love, hope and gratitude are highly linked to life satisfaction [31, 32], and negatively associated with stress [33]. Some scholars argue that four-factor virtues, namely, transcendence (e.g., love, gratitude and hope), interpersonal, openness and restraint, were preferable as they emerged as the best solution using the method of parallel analysis and the minimum average partial analysis [29]. Therefore, this study only focused on the strengths of love and gratitude and hope, which are organized under the virtue of transcendence based on the classification from Casali and others [29]. Referring to questionnaire items from Chinese Virtues Questionnaires developed by Duan and others [30], this study used the following three statements to measure love, gratitude, and hope: “There are people that I care about among my neighbors, colleagues, or classmates” (CS1), “When I look at my life, I find that there are many places to be grateful for” (CS2), and “Even in the face of challenges, I am always hopeful about the future” (CS3).

This study set social capital as the control variable. Based on Liu et al.’s (2015) research design, social capital was measured based on two dimensions: social trust and shared values, strong ties, and weak ties. The measurement of social trust and shared values consists of four items: “I feel that the neighbors in the community where I live trust each other” (SV1), “I think that the neighbors and I have almost the same concept” (SV2), “When encountering difficulties, I believe that I can ask the neighborhood committee for help” (SV3), “When I encounter difficulties, I can ask my neighbor for help” (SV4). These questions were measured on a 5-point Likert scale (strongly disagree = 1; strongly agree = 5). Strong ties were measured by the number of close friends in the community, while weak ties were measured by the number of acquaintances in the community. Other studies have confirmed that gender, age, educational attainment, marital status, annual family income [34, 35], length of residence [36], housing ownership [37], and housing area *per capita* [38, 39] affect residential satisfaction. The housing area *per capita* was measured by using housing area divided by household size. Therefore, these sociodemographic variables were also considered in this study.

### Data Analysis

The analyses of the survey data were conducted in three parts. Firstly, this study conducted a descriptive statistical analysis to demonstrate the study population characteristics. Subsequently, reliability and validity tests were conducted to evaluate the measurement of survey questionnaires; we used statistical software R to calculate Cronbach’s alpha, the most widely used objective measure of reliability; we used the statistical software AMOSS 22 to test construct validity, convergent validity and discriminant validity. Finally, linear regression analysis was conducted. Given that the independent variables of self-efficacy, character strengths, and control variables of social trust and shared value were measured by three or four items, we calculated the mean values of the measurement items for each latent variable. The final value of the dependent variable of residential satisfaction was gained by calculating the mean value of two measurement items. We used the statistical software R to run the linear regression to estimate the effects of influencing variables on residential satisfaction during the initial outbreak of the COVID-19 pandemic and subsequent self-isolation at home.

## Results

### Study Population Characteristics

Table 1 reports descriptive statistics of categorical variables. Overall, 47.7% [134/281] of the respondents were male and 52.3% [147/281] were female. With respect to marital status, 85.4% of the respondents were married and 14.6% were unmarried. The ages of the majority of respondents ranged from 45 to 54 years (55.5%), followed by the group of residents of ages 35–44 (14.6%). In terms of educational attainment, 179 respondents (63.7%) had college or undergraduate degrees. Regarding annual income, 49.5% of respondents’ annual income was reported to be less than CNY 100,000, and that of 34.9% of the respondents was reported to be between CNY 200,000–300,000; this indicates that residents living in the surveyed communities are financially well-off. In terms of *per capita* housing area, 20.3% and 19.9% of the respondents have a *per capita* housing area of 21–30 m^2^ and 31–40 m^2^, respectively; lastly, 35.6% of the respondents have a *per capita* housing area of more than 71 m^2^. The majority of respondents had lived in the surveyed community for 1–5 years. Moreover, 39.2% and 2.1% of respondents reported that approximately 1–5 cases and 6–10 cases, respectively, have been confirmed in their community.

**TABLE 1:** Descriptive statistics of categorial variables (Xiangyang, China. 2020).

Variable	Frequency (*n* = 281)	Percentage (*n* = 281)
Gender		
Male	134	47.7
Female	147	52.3
Marital status		
Unmarried	41	14.6
Married	240	85.4
Age		
15–24	18	6.4
25–34	35	12.5
35–44	41	14.6
45–54	156	55.5
Over 55	31	11.0
Educational attainment		
Middle school or below	6	2.1
High school or vocational school	60	21.4
College	179	63.7
Graduate school	36	12.8
Annual income		
Less than 100,000	139	49.5
CNY 100,000–199,999	98	34.9
CNY 200,000–299,999	27	9.6
CNY 300,000–499,999	12	4.3
CNY 500,000 or over	5	1.8
Housing area *per capita*		
11–20 m^2^	17	6.0
21–30 m^2^	56	19.9
31–40 m^2^	57	20.3
41–50 m^2^	16	5.7
51–60 m^2^	12	4.3
61–70 m^2^	23	8.2
Over 71 m^2^	100	35.6
Length of residence		
1–5	264	94.0
Over 5	17	6.0
The number of confirmed cases of COVID-19 (N_COVID-19)		
None	165	58.7
1–5	110	39.2
6–10	6	2.1

Table 2 shows the descriptive statistical analysis of the continuous variables. The average score of residential satisfaction was 4.16 (SD = 0.92), indicating that most residents were satisfied with their communities during the lockdown. However, the levels of residential satisfaction decreased with an increasing number of confirmed cases (Supplementary Material S3). The average score of transcendence, social trust and shared value, and self-efficacy was relatively high—it was 4.82 (SD = 0.46), 4.26 (SD = 0.98), and 4.25 (SD = 1.07), respectively, indicating a right-skewed distribution of these three factors. The mean value of strong and weak ties was 2.06 (SD = 1.49) and 1.47 (SD = 1.28) respectively, indicating that the self-isolation strategies minimized residents’ network size, especially for the weak ties.

**TABLE 2:** Descriptive statistics of continuous variables (Xiangyang, China. 2020).

Variable	Mean	SD	Min	Max
Self-efficacy (SE)	4.25	1.07	1.00	5.00
Social trust and shared value (SV)	4.26	0.98	1.00	5.00
Transcendence	4.82	0.46	2.67	5.00
Residential satisfaction (RS)	4.16	0.92	1.00	5.00
Strong ties	2.06	1.49	1.00	7.00
Weak ties	1.47	1.28	1.00	7.00

### Reliability and Validity Analysis

This study tested the reliability and validity of the data; the results are presented in Table 3. The Cronbach’s alpha value of each variable was higher than 0.7, indicating good reliability of the data.

**TABLE 3:** The results of the reliability test (Xiangyang, China. 2020).

Variable	Number of items	Cronbach’s α
Self-efficacy	4	0.936
Social trust and shared value	3	0.904
Transcendence	3	0.875
Residential satisfaction	2	0.914

Given that the underlying structure of the Likert scale for dependent variables, explanatory variables, and control variables has been established on prior empirical grounds, using confirmatory factor analysis (CFA) is suitable for assessing construct validity. According to Fornell and Larcker [40], convergent validity can be assessed by using three indicators: factor loading, composite reliability (CR), and average variance extracted (AVE). As shown in Table 4, the value of factor loading for each questionnaire item was over 0.7, which is higher than the acceptable value of 0.5. The value of CR was greater than 0.8, which is more than the acceptable value of 0.7. The AVE value was over 0.7, which is more than the acceptable value of 0.5. Therefore, the scale has ideal convergence validity.

**TABLE 4:** The results of confirmatory factor analysis (Xiangyang, China. 2020).

Variable	Item	Factor loading	Composite reliability (CR)	Average variance extracted (AVE)
Social trust and shared value (SV)	SV1	0.932	0.937	0.788
	SV2	0.873		
	SV3	0.872		
	SV4	0.873		
Transcendence	CS1	0.754	0.921	0.798
	CS2	0.985		
	CS3	0.925		
Self-efficacy (SE)	SE1	0.795	0.882	0.714
	SE2	0.868		
	SE3	0.870		
Residential satisfaction (RS)	RS1	0.853	0.919	0.851
	RS2	0.987		

To test discriminant validity, we calculated the square root of the AVE. If the levels of the square root of the AVE for each latent variable are greater than the correlation involving the latent variable, then the scale has excellent discriminant validity [40]. As shown in Table 5, the AVE’s square root for the variables of self-efficacy, character strengths, social trust and shared value, and residential satisfaction are all over 0.8 (0.845, 0.893, 0.888, and 0.922, respectively), which are higher than the correlations involving the latent variable; Thus, the scale has excellent discriminant validity.

**TABLE 5:** The results of discriminant validity (Xiangyang, China. 2020).

	Self-efficacy	Character strengths	Social trust and shared value	Residential satisfaction
Self-efficacy	**0** **.** **845**			
Transcendence	0.279	**0.893**		
Social trust and shared value	0.615	0.554	**0.888**	
Residential satisfaction	0.484	0.474	0.796	**0.922**

The bold value indicates the square root of the AVE.

### Regression Analysis

Prior to regression analysis, we used the statistical software R to calculate the variance inflation factor (VIF) to detect the multicollinearity. The results revealed that all the VIF values are less than 3, indicating a moderately low correlation between the variables. Therefore, the calculated regression coefficient estimates are unbiased and valid. The regression analysis results are shown in Table 6. Model 1 included only the control variables. Model 2 included control and independent variables. Model 3 included the interaction effect between self-efficacy and the number of confirmed COVID-19 cases. In terms of the explanatory variable, the statistical outcomes of Model 2 revealed that the number of confirmed COVID-19 cases negatively impacted residential satisfaction. This implies that the higher the number of confirmed cases in the community, the lower the residential satisfaction. The statistical outcome in Model 2 revealed that self-efficacy does not directly exert a significant impact on residential satisfaction. However, the statistical outcome in Model 3 revealed that self-efficacy moderated the relationship between the number of confirmed COVID-19 cases and residential satisfaction.

**TABLE 6:** Influencing factors on residential satisfaction during COVID-19 (Xiangyang, China. 2020).

Variable	Model 1			Model 2			Model 3		
	Standardized coefficient (beta)	SE	CI	Standardized coefficient (beta)	SE	CI	Standardized coefficient (beta)	SE	CI
Constant	1.299*	0.515	0.285 to 2.314	1.008	0.541	−0.058 to 2.073	2.4655***	0.664	1.158 to 3.772
Gender (ref. = Male)									
= Female	−0.086	0.070	−0.224 to 0.052	−0.054	0.063	−0.178 to 0.069	−0.058	0.061	−0.179 to 0.063
Marital status (ref = unmarried)									
= Married	0.965***	0.169	0.631 to 1.298	1.041***	0.152	0.741 to 1.341	1.089***	0.149	0.795 to 1.384
Housing ownership (ref. = Renter)									
= Homeowner	−0.066	0.073	−0.209 to 0.077	−0.023	0.065	−0.151 to 0.105	0.008	0.064	−0.118 to 0.134
Community (ref. = TXH)									
=ZGC	−0.020	0.121	−0.26 to 0.219	−0.076	0.109	−0.290 to 0.138	−0.113	0.107	−0.323 to 0.098
=SJQ	−0.104	0.080	−0.261 to 0.053	−0.107	0.071	−0.246 to 0.033	−0.106	0.069	−0.242 to 0.031
=WF	0.100	0.124	−0.144 to 0.343	0.098	0.111	−0.121 to 0.316	0.092	0.109	−0.122 to 0.306
=TFS	−0.211	0.299	−0.801 to 0.378	−0.308	0.271	−0.841 to 0.225	−0.364	0.265	−0.885 to 0.158
Age	0.231***	0.053	0.126 to 0.335	0.252***	0.051	0.150 to 0.353	0.265***	0.050	0.165 to 0.364
Educational attainment	−0.178*	0.071	−0.317 to −0.038	−0.176**	0.063	−0.301 to −0.052	−0.213***	0.063	−0.336 to −0.090
Annual income	−0.006	0.045	−0.094 to 0.081	−0.043	0.042	−0.126 to 0.039	−0.016	0.042	−0.098 to 0.066
Housing area *per capita*	0.049**	0.018	0.014 to 0.085	0.043*	0.016	0.010 to 0.075	0.048**	0.016	0.016 to 0.079
Length of residence	−0.356*	0.168	−0.687 to −0.025	0.003	0.159	−0.309 to 0.316	−0.136	0.160	−0.451 to 0.179
Social trust and shared value	0.668***	0.040	0.589 to 0.748	0.636***	0.050	0.538 to 0.735	0.589***	0.051	0.490 to 0.689
Weak ties	0.062*	0.029	0.005 to 0.120	0.022	0.027	−0.032 to 0.076	0.032	0.027	−0.021 to 0.086
Strong ties	0.044	0.027	−0.010 to 0.097	0.009	0.025	−0.040 to 0.058	0.018	0.024	−0.031 to 0.066
Transcendence				0.191*	0.085	0.024 to 0.358	0.279***	0.086	0.109 to 0.449
N_COVID-19				−0.555***	0.067	−0.687 to −0.424	−0.488***	0.068	−0.621 to −0.355
Self-efficacy				−0.076	0.043	−0.160 to 0.008	−0.123**	0.044	−0.209 to −0.037
N_COVID-19*Self-efficacy							0.217***	0.060	0.099 to 0.334
F	29.5			35.18			35.58		
R^2^	0.625			0.707			0.721		
△R^2^	0.604			0.687			0.701		

Note:*p < 0.05, **p < 0.01, and ***p < 0.001. N_COVID-19 means the number of confirmed cases.

As for character strengths, this study only focuses on the strengths of love, gratitude and hope that are highly associated with wellbeing and are organized under the virtue of transcendence based on the classification of Casali and others [29]. The coefficient of transcendence is positive, indicating that individuals with high levels of transcendence are more likely to have high levels of residential satisfaction. Strengths of love, gratitude, and hope both have positive correlations with residential satisfaction (Supplementary Material S4).

The control variables of social trust and shared values, marital status, educational attainment, age, and housing area *per capita* in Models 2 and 3 significantly impacted residential satisfaction. The statistical results of Models 2 and 3 revealed that the impacts of weak ties and strong ties on residential satisfaction were insignificant during quarantine. Taking the unmarried group as a reference, the effect of marriage on enhancing residential satisfaction was strong in the three models, indicating that married people have higher residential satisfaction than that of unmarried people. The coefficient of educational attainment is negative, indicating that the higher the educational attainment, the lower the degree of residential satisfaction during the pandemic. This study posits that older residents have higher residential satisfaction than young people during quarantine. The coefficient of housing area *per capita* is positive, indicating that residents have higher levels of residential satisfaction when the housing area *per capita* is larger during quarantine.

## Discussion

This study chose Xiangyang City, one of the high-risk districts in Hubei Province, to explore the factors influencing residential satisfaction during the initial stage of the COVID-19 pandemic. Based on 281 observations from five communities, the statistical outcomes revealed that the explanatory variable of character strengths and the number of confirmed COVID-19 cases had a significant influence on residential satisfaction during quarantine. Peterson and Seligman [26] have argued that character strengths can determine how individuals cope with adversity in stressful situations. Individuals with high levels of character strengths are able to decrease mental distress when confronted with an unpredictable threat [19, 20]. As mentioned above, this study focused on the virtue of transcendence comprising the strengths of love, gratitude and hope. The results of virtue of transcendence imply that residents in Xiangyang City can transcend the current distress situation. The strengths of love and gratitude are directly related to the positive emotional states, which can decrease COVID-19 related stress [33] and increase mental health during the COVID-19 pandemic lockdown [28]. Individuals with strengths of hope often have a positive outlook on the future and are always strong and resilient when facing negative life events [41]. Such strength seems vital for residents to cope with anxiety under the stressful COVID-19 pandemic [42]. This finding also echoes Karatas and others’ argument that residents with more hopeful lives have a higher life satisfaction [43]. Under the lockdown policy, residents in Xiangyang City with strengths, such as gratitude (being aware of and thankful for the good things), hope (expecting the best in the future and working to achieve it), and love (valuing close relations with others, in particular, those in which sharing and caring are reciprocated), have the ability to improve mental health and promote subjective wellbeing. This finding is consistent with the conclusion conducted under normal conditions that the strengths of love, hope and gratitude are highly linked to life satisfaction [31, 32].

The number of confirmed COVID-19 cases in the community located in Xiangyang City was found to be negatively related to residential satisfaction. An increase in the number of confirmed cases enhanced Chinese residents’ anxiety and fear of the virus. Fear and stress were also high during the initial outbreak period in the absence of effective medicines and vaccines [2].Some studies found that social isolation in the wake of the COVID-19 pandemic significantly predicts poor mental health, such as psychological distress [44].Psychological distress is negatively related to residential satisfaction [21,45]. Additionally, communities with confirmed cases usually adopted stricter restrictions regarding social contact and activities in China. Some studies revealed that a lack of social support and a change in lifestyle may be more threatening to individuals’ wellbeing than the risk of infection [5]. In addition, some residents and their neighbors became mutually suspicious to avoid infection at the initial stage of the COVID-19 pandemic in Xiangyang City.

As a psychological factor, although self-efficacy does not directly impact residential satisfaction, this study found that it moderated the relationship between the number of confirmed COVID-19 cases and residential satisfaction. Considering that COVID-19 is an infectious virus with relatively high mortality at the initial stage of the outbreak, Chinese residents might have had low inner beliefs about overcoming the virus. Therefore, the direct positive impact of self-efficacy on residential satisfaction might be traded off by psychological distress (e.g., anxiety and panic). When Chinese residents have a high level of self-efficacy, the negative impact of confirmed COVID-19 cases on residential satisfaction was weakened. Individuals with a higher sense of self-efficacy are psychologically stronger in coping with COVID-19. Self-efficacy can reduce the psychological impact of the pandemic on residents and mitigate the panic and anxiety triggered by the spread of COVID-19. Therefore, self-efficacy can mitigate the negative impact of confirmed COVID-19 cases on residential satisfaction. These moderating effects corroborated that self-efficacy was a protective factor against anxiety symptoms in China. In contrast, the variable of confirmed COVID-19 cases also moderated the relationship between self-efficacy and residential satisfaction (Supplementary Material S5). This implies that the large number of confirmed cases reported in the communities strengthened the impact of self-efficacy on residential satisfaction. As mentioned above, self-efficacy does not directly impact residential satisfaction; however, this relationship occurs in communities without confirmed COVID-19 cases. When Chinese residents live in communities with increasing numbers of confirmed COVID-19 cases, the positive effects of self-efficacy on residential satisfaction are also strengthened.

The need for social connection is a core human characteristic [46]. This study also explored the impact of social capital on residential satisfaction. This variable has seldom been analyzed by psychologists, but it must be noted that social distancing strategies have changed residents’ social capital unprecedently during self-isolation at home. Therefore, this study added social capital as the control variable. The statistical outcomes in the small- and full-model specifications reveal that social trust and shared value positively impacted residential satisfaction. When Chinese residents trust the surrounding environment and have shared values with their neighbors, they have a sense of security and belonging. A high level of security can improve residential satisfaction. Additionally, the effect of social trust and shared value on residential satisfaction was higher than that of character strengths in Xiangyang City. This finding implies that Chinese residents paid more attention to the relationship and trust among neighbors during the lockdown. Community trust and shared values may have helped decrease the nervous atmosphere during the COVID-19 outbreak. The outcomes also reveal that social isolation makes strong ties and weak ties become insignificant in China. Weak ties between different people can help these people gain more information, which is helpful for their integration into communities [47]. Studies conducted during normal conditions (before COVID-19 pandemic) found that strong and weak ties are associated with received social support, and thus conferring essential benefits for better health and wellbeing [48]. However, other studies also posit that the function of social capital to increase life satisfaction depends on the context [49]. Our survey was conducted in the context of the first wave of the COVID-19 pandemic in China. Fear and stress were also high during this period because of the lack of effective medicine and vaccine provision at that time [2]. Social isolation and limited physical activity, on the other hand, was considered to protect residents from being infected. Some researchers found that death anxiety was positively associated with behavioral compliance with unprecedented restrictions [50]. Therefore, it may be explained that Chinese residents accepted quarantine strategies to cope with the spread of the virus at the cost of losing strong and weak ties.

The sociodemographic variables of marriage, age, educational attainment, and housing area *per capita* were also found to have a significant impact on residential satisfaction. Band-Winterstein and Manchik-Rimon [51] found that older never-married childless singles experience a continuum between solitude and loneliness before the pandemic. Loneliness predicts depressive mood, leading to a low level of belongingness [52]. Other studies also revealed that male widowhood was associated with lower levels of belongingness [53]. Those findings seem to uncover that married residents have a stronger sense of belonging than unmarried residents. However, the influencing mechanisms may have changed during quarantine. The mental support that married individuals can gain from their spouses played a vital role in decreasing their fear and feelings of panic during the rapid spread of the virus in China. The fear of COVID-19 was found negatively correlated with residential satisfaction [14]. Our study found that the higher the educational attainment, the lower the degree of residential satisfaction during the pandemic in China. This finding aligns with existing studies showing that years of education are negatively associated with life satisfaction [54]. However, this relationship was corroborated under the particular conditions of COVID-19. Individuals with higher educational attainment always expressed a higher level of fear and panic feelings toward the virus [55]. Psychological distress is negatively associated with residential satisfaction. In addition, some scholars found that while highly educated individuals have a high threat appraisal for COVID-19, they are unwilling to adopt preventive behaviors of self-isolation [55]. The self-isolation policy might bring inconvenience to their daily work and life, contributing to a low level of residential satisfaction in China.

Unlike the findings of Montero and others [56], who argued that older adults are more likely to have a lower level of life satisfaction based on the data collected from Chile for the years 2011 and 2013, this study posits that older residents have higher residential satisfaction than young people during quarantine in China. This contradictory result may be explained by different cultural and pandemic contexts. The data in this study were collected during the initial stages of the COVID-19 pandemic. Older adults are more likely to experience panic and feel fear toward COVID-19 [57], because COVID-19 mortality and fatality rates are associated with old age [58]. Other studies have posited that, although older adults have negative emotions during the pandemic, the pandemic can also have upside and hence positive impacts on residential satisfaction [59]. Older adults are more likely to engage in preventive behaviors due to fear of COVID-19 [55]. Therefore, Chinese older adults might be more willing to accept the protective strategy of self-isolation, leading to higher residential satisfaction during the self-isolation period. The statistical outcome revealed that Chinese residents have higher levels of residential satisfaction when the housing area *per capita* is larger. The value of housing area *per capita* directly determines how much space residents can engage in family activities at home. Boredom has been reported during the pandemic. A larger housing area will provide more living space and produce different living experiences for residents in China, leading to an increase in satisfaction during the self-quarantine period.

This study has several limitations that should be considered when interpreting the results. First, the data collected in Xiangyang may not be representative. This study used a convenience sample instead of a random sample. Most respondents had a bachelor’s degree, which led to uneven distribution of the sample by educational attainment. Additionally, the data were collected through the Internet during the quarantine, but the online survey method may have excluded respondents who may be less comfortable navigating an online survey, such as the group of elderly people. The proportion of elderly people (over 55 years old) is merely counting for 11%. Therefore, the generalizability of our findings is limited. Future studies could use a random sampling approach to include diverse samples. Second, the measures for psychological variables were self-reported by respondents, although this was prevalent among many studies conducted during the COVID-19 pandemic. Self-reported methods are subject to respondents’ recall biases. Future studies could examine the variables by using alternative methods for data collection, such as in-depth interviews. Third, due to the limited time and resources, this study employed a cross-sectional approach to collect data, but it was not possible to infer causality from the findings. Future studies should use a longitudinal design, such as the cohort study, to explore the causal relationship between the study variables.

The findings of this study have several practical implications. We suggest that residents in the community be encouraged to break the barriers between neighbors and establish a community trust system by improving communication and interaction among neighbors. Social support can decrease depression symptoms [60]. Frequent participation in community activities has a positive relationship with residential satisfaction. Furthermore, local governments should provide funding to support regular psychological health lectures. Improving residents’ psychological self-efficacy can enhance their response to unpredictable threats, which can decrease the impact of panic and fear triggered by unknown risk factors on residential satisfaction. Residents should be given the opportunity to develop a “personal resilience plan,” that involves identifying and anticipating response challenges. Finally, the government should suggest ways to cope and deal with physical and mental fatigue to enhance personal character strengths. Some studies have found that, despite character strengths being stable, they are also malleable [26]. Recovered individuals with traumatic experiences often demonstrate elevated character strengths. Similarly, some studies have revealed that the COVID-19 pandemic promotes character growth, and that preventive character strengths interventions are necessary for residents to develop resilience [61]. Thus, it is necessary to encourage residents to develop character strengths under the high-risk society.

## Data Availability

The data that support the findings of this study are available from the first author based on reasonable request.
