# Supplementary material for: Determinants of Residential Satisfaction During the Initial Stage of the COVID-19 Pandemic: The Case of Xiangyang City, China
Source: Int J Public Health. 2023 Nov 28;68:1606016. doi: 10.3389/ijph.2023.1606016 (PMC10713804; doi:10.3389/ijph.2023.1606016)
Supplement: Supplementary file 1 [file DataSheet1.docx]

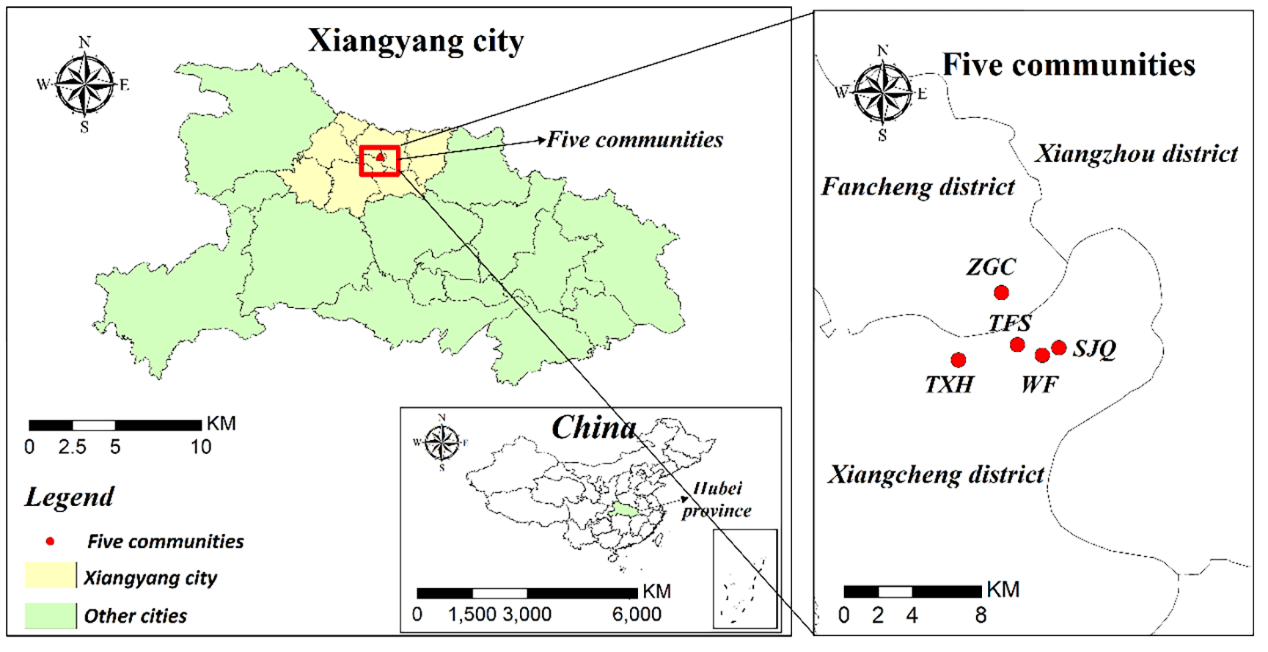


Supplementary Material S1: The location of field sites (Xiangyang, China. 2020).


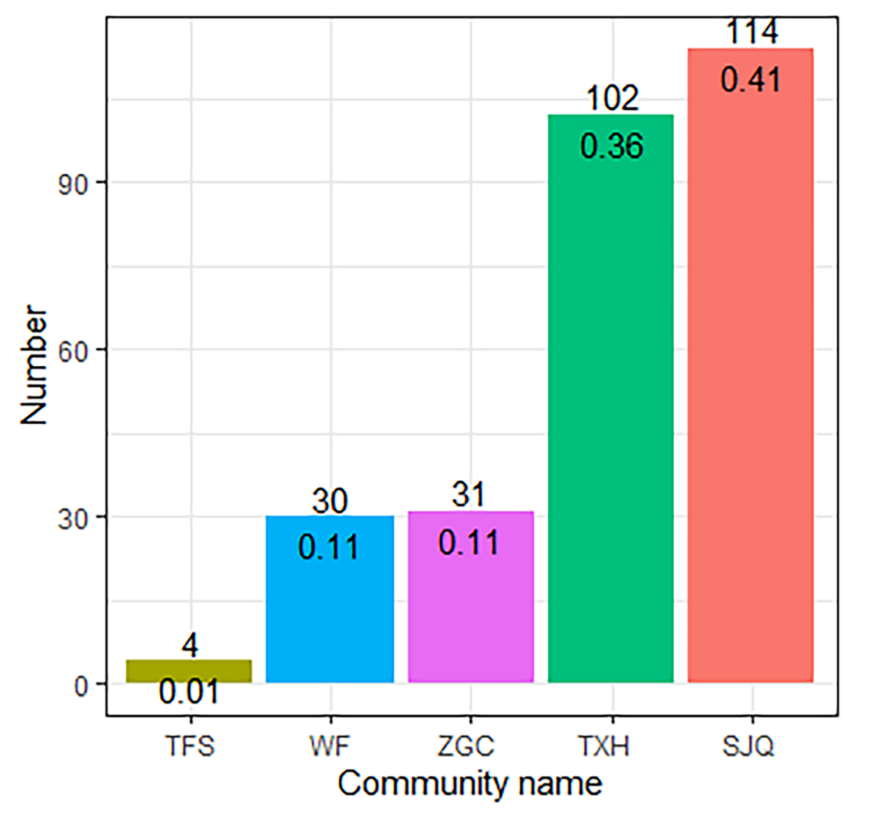


Supplementary Material S2: The distribution number of surveyed communities (Xiangyang, China. 2020).


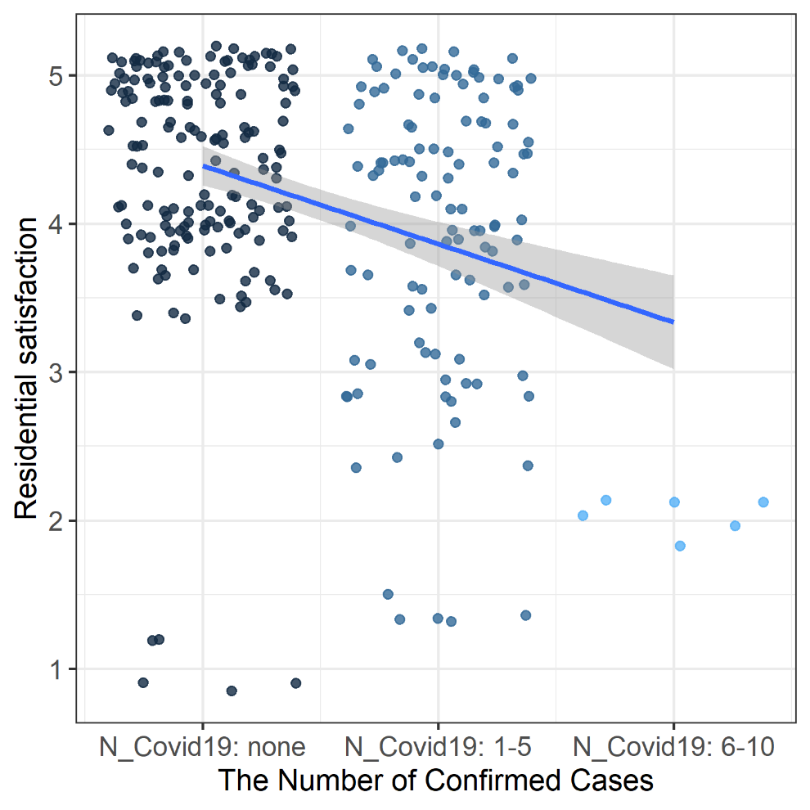


Supplementary Material S3: The relationship between residential satisfaction and number of confirmed cases (Xiangyang, China. 2020).


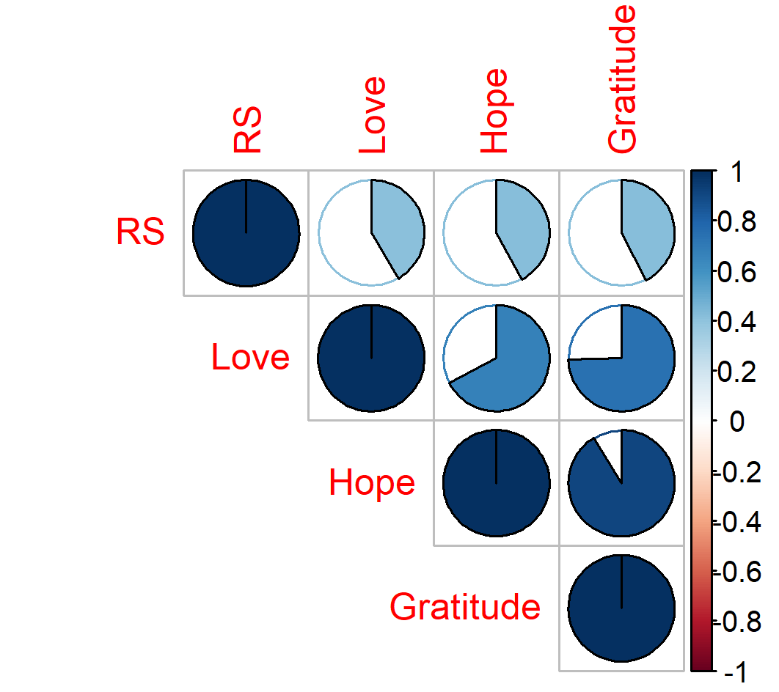


Supplementary Material S4: The correlation between residential satisfaction and character strengths (Xiangyang, China. 2020).tiff


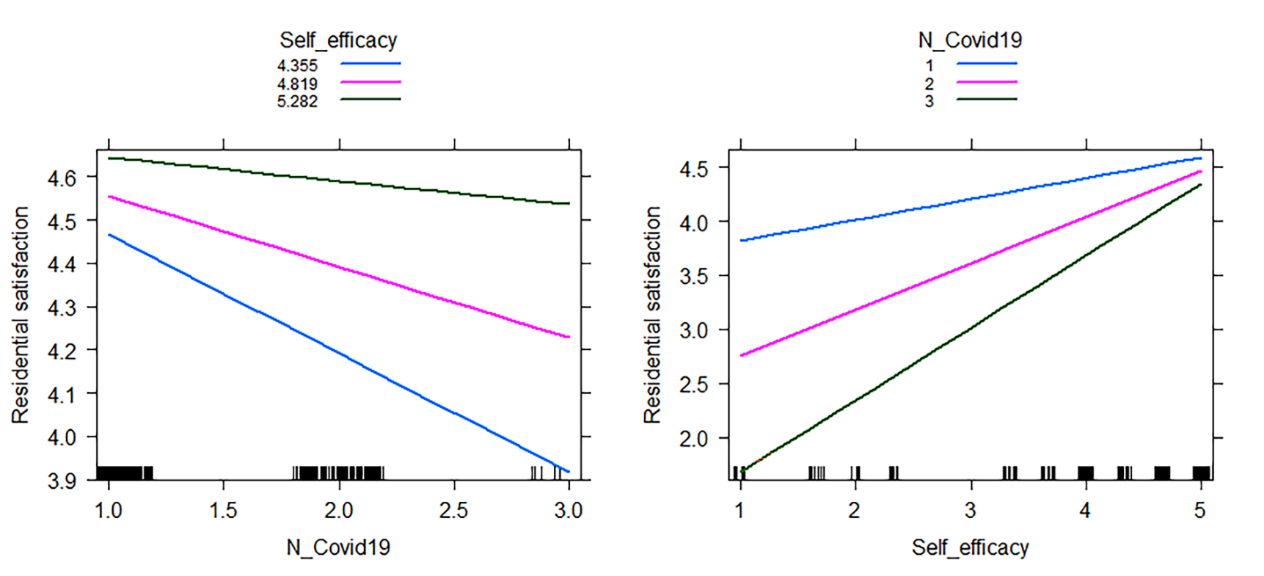


Supplementary Material S5: The moderating effect of self-efficacy and number of confirmed cases (Xiangyang, China. 2020).
